# Supplementary material for: A randomised controlled trial of Pre-Operative Oncotype DX testing in early-stage breast cancer (PRE-DX study) – Study protocol
Source: PLoS One. 2024 Mar 15;19(3):e0300339. doi: 10.1371/journal.pone.0300339 (PMC10942020; doi:10.1371/journal.pone.0300339)
Supplement: S1 Protocol — (PDF) [file pone.0300339.s002.pdf]

PRE-OPERATIVE ONCOTYPE DX TESTING:

A DECISION IMPACT STUDY

**FULL PROTOCOL TITLE**	Pre-Operative Oncotype DX testing: A decision impact study
**SHORT TITLE**	PRE-DX Study
**PROTOCOL VERSION**	V2.2
**HHTU REFERENCE NUMBER**	P114
**REC REFERENCE NUMBER**	22/LO/0421
**ISRCTN REFERENCE NUMBER**	ISRCTN14337451
**CHIEF INVESTIGATOR**	Mr Henry Cain MBChB hon, FRCS, MD<br>Consultant Oncoplastic Breast Surgeons<br>Level 4 Leazes Wing<br>Royal Victoria Infirmary<br>Newcastle Upon Tyne<br>NE1 4LP
**CO-INVESTIGATOR**	Mr Andrew Pieri MBBS FRCS<br>Consultant Oncoplastic Breast Surgeons<br>Level 4 Leazes Wing<br>Royal Victoria Infirmary<br>Newcastle Upon Tyne<br>NE1 4LP
**DATE**	04.07.2023

KEY CONTACTS

Sponsor	The Newcastle Upon Tyne Hospitals NHS Foundation Trust (NuTH)<br><br>Newcastle Joint Research Office<br>Level 1, Regent Point<br>Regent Farm Road<br>Gosforth<br>Newcastle upon Tyne<br>NE3 3HD<br><br>tnu-tr.sponsormanagement@nhs.net
Authorised protocol and amendment signatories (Investigator, Sponsors)	Mr Henry Cain, Chief Investigator<br>NUTH NHS Trust, Sponsor<br>Newcastle Joint Research Office<br>First floor, Regent Point<br>Gosforth<br>NE3 3HD
Collaborative Trials Units and Institutions	Hull Health Trials Unit<br>3rd Floor Allam Medical Building,<br>University of Hull<br>Hull HU6 7RX
Collaborators	Professor Sarah Pinder<br>Professor of Breast Pathology<br>King's College London<br>Comprehensive Cancer Centre<br>London SE1 9RT<br>**T** 020 7188 4260<br>sarah.pinder@kcl.ac.uk<br><br>Dr Sarah Reynia<br>Medical Advisor<br>Exact Sciences<br>Suite 1 – Scott House<br>London SE1 7LY<br>**T** 020 3031 8087<br>sreynia@exactsciences.com

	Dr Judith Cohen,<br>Director Hull Health Trials Unit<br>Allam Medical Building<br>University of Hull<br>Hull HU6 7RX<br>**T** 01482 463382<br>judith.cohen@hyms.ac.uk<br><br>Mr James Harvey<br>Consultant Breast Surgeon<br>Manchester University FT<br>Nightingale Centre, Southmoor Road<br>Manchester M23 9LT<br>**T** 0161 2914436<br>james.harvey@mft.nhs.uk<br><br>Professor Carlo Palmieri<br>Consultant Medical Oncologist<br>Clatterbridge Cancer Centre<br>NHS Foundation Trust<br>65 Pembroke Place<br>Liverpool L7 8YA<br>c.palmieri@liverpool.ac.uk<br><br>P.G. Roy<br>Consultant Oncoplastic Breast Surgeon<br>NIHR Oxford University Hospital<br>Oxford OX3 9DU<br>**T** 01865 220935<br>pankaj.roy@ouh.nhs.uk<br><br>Dr Mark Verrill<br>Northern Centre for Cancer Care<br>Freeman Road<br>Newcastle upon Tyne<br>NE7 7DN<br>**T** 0191 2138475<br>mark.verrill@nhs.net
Trial Management and monitors	HHTU Trial Manager<br>Dr Matthew Northgraves<br>Hull Health Trials Unit<br>Allam Medical Building<br>University of Hull

	Hull HU6 7RX<br>Matthew.Northgraves@hyms.ac.uk<br><br>HHTU Trial Coordinator:<br>Paul Bradley<br>Hull Health Trials Unit<br>Allam Medical Building<br>University of Hull<br>Hull HU6 7RX<br>Paul.Bradley@hyms.ac.uk
Trial Statistician(s)	Dr Chao Huang<br>Senior Lecturer in Statistics<br>Allam Medical Building<br>University of Hull<br>Hull HU6 7RXT<br>**T** 01482 463281<br>chao.huang@hyms.ac.uk
Emergency contact in the event of the CI becoming unavailable	Mr Andrew Pieri MBBS FRCS<br>Consultant Oncoplastic Breast Surgeons<br>Level 4 Leazes Wing<br>Royal Victoria Infirmary<br>Newcastle Upon Tyne<br>NE1 4LP

Protocol amendments summary

State "none" if no amendments			
	Version number	Date	Provide reason for the amendment<br>State which sections of the protocol were replaced, added or deleted
N/A - Response to REC comments	1.1	27.06.2022	Section 4.1.1. Update of inclusion criteria<br>Section 4.3.2. Update to withdrawal section
Clarification of primary outcome and primary endpoint	2.0	13.01.2023	Cover page updated<br>Trial Summary: Including additional sites<br>Study design: Including additional sites<br>Section 3.2: Clarification to primary outcome<br>Section 3.3: Clarification to first secondary outcome<br>Section 3.4: Updated study flow chart

			Section 4.1.1: Removal of size requirement for Grade 3 tumours and correction of typographical error – replaced > with ≥<br>Section 4.2: Clarification for approach process when waiting for test results to confirm eligibility including introduction of approach email<br>Section 4.3: Clarification of postal consent process<br>Section 5.1: Clarification of process when both follow questionnaires due on the same day<br>Update to table of data collection<br>Appendix A – Updated to replicate end point definition
Clarification eligibility of patients receiving bridging therapy and on ordering samples for these participants	2.1	24.03.2023	Section 3.1: Clarification on process when a participant has received bridging therapy prior to surgery<br>Section 3.2: Clarification of primary outcome when endocrine treatment has been received<br>Section 4.1.2: Clarification on exclusion criterion 'Planned for neoadjuvant systemic therapy'<br>Section 5.2: Clarification that it is the first post-operative appointment date used for the post-operative follow-up questionnaire.
Rewording to primary outcome and correction to archiving section.	2.2	04.07.2023	Section 3.2: Rewording to the primary outcome to ensure touchpoints from diagnosis to the offer of adjuvant treatment in captured.<br>Section 3.3: Additional secondary outcome of time between diagnosis and start of adjuvant treatment added<br>Section 10: 'and duration' removed from section as not possible as not being collected.<br>Section 16: Archiving corrected to 5 years from 10 years to reflect the IRAS form and PIS.

ABBREVIATIONS

APR	Annual Progress Report
CI	Chief Investigator
CRF	Case Report Form
CTIMP	Clinical Trial of an Investigational Medicinal Product
EDC	Electronic Data Capture
ER+	Oestrogen Receptor Positive
FNA	Fine Needle Aspiration
GCP	Good Clinical Practice
GP	General Practitioner
GDPR	General Data Protection Regulation
HADS	Hospital Anxiety Depression Score
HHTU	Hull Health Trials Unit
HRUQ	Health Resource Utilisation Questionnaire
ICF	Informed Consent Form
ISF	Investigator Site File
MDT	Multi-disciplinary Team
NHS R&D	National Health Service Research & Development
NRES	National Research Ethics Service
R&D	Research & Development
PI	Principal Investigator
PIS	Participant Information Sheet
PPE	Personal Protective Equipment
RCC	REDCap Cloud (Electronic Database)
RCT	Randomised Control Trial
REC	Research Ethics Committee
RGF	Research Governance Framework
RS	Recurrence Score
SDV	Source Data Verification
SOP	Standard Operating Procedure
TMF	Trial Master File
TMG	Trial Management Group
TSC	Trial Steering Committee
UoH	University of Hull

TABLE OF CONTENTS

TRIAL SUMMARY ... 9

1.0 BACKGROUND ... 9

2.0 AIMS AND OBJECTIVES ... 10

3.0 STUDY DESIGN ... 10

3.1 INTERVENTIONS ... 10

3.2 PRIMARY OUTCOME ... 11

3.3. SECONDARY OUTCOMES ... 11

4.0 RECRUITMENT PROCESS ... 14

4.1 ELIGIBILITY ... 14

4.1.1. INCLUSION CRITERIA ... 14

4.1.2 EXCLUSION CRITERIA ... 14

4.2 IDENTIFICATION AND APPROACH ... 14

4.3 INFORMED CONSENT ... 15

4.3.1 PARTICIPANTS UNABLE TO GIVE CONSENT ... 16

4.3.2 WITHDRAWAL OF PARTICIPANTS ... 16

4.4 ENROLMENT AND RANDOMISATION ... 17

5.0 DATA COLLECTION ... 17

5.1 STUDY VISIT SCHEDULE ... 17

5.2 DATA COLLECTION METHODS ... 18

6.0 SAMPLE COLLECTION ... 20

7.0 MONITORING ... 20

8.0 SAFETY REPORTING ... 21

9.0 STUDY OVERSIGHT RESPONSIBILITIES ... 21

9.1 DAY-TO-DAY TRIAL MANAGEMENT ... 21

9.2 TRIAL MANAGEMENT GROUP (TMG) ... 21

10.0 COST IMPACT ANALYSIS ... 22

11.1 SAMPLE SIZE: ... 22

11.2 STATISTICAL ANALYSIS: 22

12.0 DATA SYSTEMS 23

13.0 DATA ACCESS 24

14.0 QUALITY ASSURANCE AND ETHICAL CONSIDERATIONS 24

14.1 RESEARCH ETHICS COMMITTEE (REC) 25

14.2 REGULATORY COMPLIANCE 25

14.3 PROTOCOL COMPLIANCE 25

14.4 SERIOUS BREACHES 25

14.5 ETHICAL CONSIDERATIONS 26

15.0 CONFIDENTIALITY 26

16.0 ARCHIVING 27

17.0 STATEMENT OF INDEMNITY 27

18.0 STUDY ORGANISATIONAL STRUCTURE 27

19.0 PUBLICATION POLICY 28

20.0 REFERENCES 28

TRIAL SUMMARY

The Oncotype DX® test is a prognostic and predictive genomic assay, validated and approved for use in early Oestrogen receptor positive (ER+) breast cancer to guide adjuvant systemic treatment. The Oncotype DX® test is performed following surgical treatment of breast cancer to more accurately determine patients who benefit from additional chemotherapy treatment. This multi-centre prospective randomised controlled trial (RCT) aims to ascertain the impact on the patient management pathway of performing the Oncotype DX Breast Recurrence Score® test on the pre-operative diagnostic core biopsy (intervention), as opposed to requesting the test on the post-operative excision specimen as it is in the current usual patient pathway. The study will analyse the effectiveness of the intervention arm pathway, as measured in patient touch points and the time from diagnosis to being offered adjuvant cancer therapy. The study will also explore if there is any alteration in the sequencing of treatments (e.g. use of neo-adjuvant strategies) in the intervention arm.

The study will recruit 330 participants from up to 25 sites across the UK.

1.0 BACKGROUND

Oncotype DX® is a prognostic and predictive genomic assay validated and approved by NICE for the use in early Oestrogen receptor positive (ER+) breast cancer to guide adjuvant systemic treatment [1]. The Recurrence Score® (RS®) result guides recommendations made to the patient regarding the requirement for adjuvant chemotherapy in addition to adjuvant endocrine therapy [2]. The Oncotype DX® test is currently performed on the operative specimen following surgical treatment of the breast cancer. This test more accurately determines the group of patients who benefit from the addition of chemotherapy. Using this test approximately 3/10 patients are shown to require chemotherapy.

It has been shown to be both technically possible and accurate to perform the Oncotype DX® test on the diagnostic core biopsy of the breast cancer prior to the surgical tumour excision procedure [3, 4], improving the timeliness of starting adjuvant treatment [5, 8]. Having the information provided by the Recurrence Score® results in the pre-operative setting would therefore be advantageous to the treating clinician and the patient. The treating clinician would be able to more accurately counsel the patient with regards to their treatment pathway and account for this in planning the patient's care. This would not only empower the patient with regards to the expectations of upcoming treatments but also expedite those treatments by eliminating the inherent delay in waiting for the RS results on the surgical excision specimen. In addition, the pre-operative knowledge regarding the type of oncological input required in the adjuvant setting will also allow the streaming of follow up appointments and mitigate against unnecessary and costly outpatient attendances.

2.0 AIMS AND OBJECTIVES

This study aims to compare the standard patient pathway (control arm), where the Oncotype DX® test is requested on the post-operative specimen with requesting the test on the pre-operative diagnostic core biopsy (intervention arm). The initial planned analyses will add to the evidence base of the patient management pathway of performing the Oncotype DX® test on the pre-operative diagnostic biopsy.

Aim: To assess the impact on patient management by the availability of the RS® results from the core biopsy in the pre-operative setting.

Primary hypothesis: The availability of the RS® results from the core biopsy in the pre-operative setting streamlines the patient management pathway

Secondary hypothesis: The availability of the RS® results from the core biopsy in the pre-operative setting reduces healthcare utilisation, improves patient experience and decreases the time to the offering of adjuvant cancer therapy.

3.0 STUDY DESIGN

A multi-centre, prospective parallel group randomised controlled trial with unequal random allocation (2:1; Intervention:Control) comparing the impact on the patient management pathway of performing the Oncotype DX® test on the diagnostic core biopsy in the pre-operative setting (intervention) as opposed to the surgical excision biopsy (control)[#]. The primary outcome and end point will be number of touchpoints between the treating team and the participant from the initial patient approach to the start of adjuvant cancer treatment. The study will recruit 330 participants from up to 25 sites across the UK.

3.1 INTERVENTIONS

For participants in the Standard arm, standard practice will be followed at each site for ordering and acting on the Recurrence Score® (RS®) result. The Oncotype DX® test is performed on excision samples obtained during surgery[#].

[#]Participants receiving endocrine bridging therapy are eligible for the study provided the reason is that there has been a delay in planned surgery. Patients prescribed neo-adjuvant endocrine or chemotherapy for the purpose of downstaging are **<u>not</u>** permitted. In the event of the participant having received endocrine bridging therapy prior to surgery, as per routine practice, the use of the core biopsy for testing is permitted in the standard arm provided it is still ordered in the post-operative setting.

For participants in the Intervention arm, the Oncotype DX® test will be performed on the core biopsy that was taken at the time of diagnosis and the results of the Oncotype DX® test will be available to be discussed at the post-operative MDT meeting. The discussion will support decision-making on treatment options.

A failure of RS scores on the diagnostic biopsy sample would mean the test result being unavailable to support adjuvant treatment decisions for participants in the Intervention Arm. In the event of a failure on the diagnostic biopsy sample, the excision sample will be retested as per the surgical excision biopsy group. They will continue in the study in their original allocated group, being included in the primary intention to treat analysis.

Planned surgery will not be delayed if RS® results are unavailable and any change to the treatment recommendation will be recorded and discussed with the participant at an additional routine clinic appointment if required. If there is no change to the treatment recommendation, it will be left to the Centre's discretion whether to discuss the results with the patient before surgery but intentions of the centre will be recorded. If the event the decision is made to change the treatment pathway with the patient subsequently receiving neo-adjuvant or endocrine therapy prior to surgery, the clinical touchpoints, HADS and HRUQ would no longer be collected but the participants would be followed up until completion of treatment for final post-operative pathology.

In both arms of the study, the interpretation of the RS® result and treatment recommendations will be at the discretion of the MDT and treating clinicians following national guidelines.

3.2 PRIMARY OUTCOME

- Number of participant touch points defined as a participant-clinician interaction (outpatient appointment or equivalent) between the treating team and the participant from the participant diagnosis (defined as the date of attendance at diagnostic clinic) to the offer and prescription* of the first adjuvant cancer treatment (see Appendix A for definitions). In the event of the participant declining further treatment the date of offer should be used.

For those participants receiving bridging therapy, the date of the confirmation of adjuvant systemic therapy (endocrine or chemotherapy) will be used.

*Defined as the date prescription is documented as being issued in the clinic appointment **or** letter sent to GP to request prescription **or** treatment entered into Chemocare or equivalent.

3.3. SECONDARY OUTCOMES

- Time in days between the participant diagnosis (defined as the date of attendance at diagnostic clinic) and the offer and prescription of the first adjuvant cancer treatment (chemotherapy, radiotherapy or endocrine therapy)

- Time in days between the participant diagnosis (defined as the date of attendance at diagnostic clinic) and the start of the first adjuvant cancer treatment (chemotherapy, radiotherapy or endocrine therapy)

- Alteration in recommended treatment sequence. This would be defined as a when a participant in the intervention arm, as a result of the RS results from the core biopsy, receives neo-adjuvant treatment rather than proceeding directly to surgery. The clinical touchpoints, HADS and HRUQ would no longer be collected but the participants would be followed up until completion of treatment for final post-operative pathology.

- Participant reported anxiety score HADS score at the post-operative result clinic and following the offer of adjuvant treatment

- Health system cost impact analysis comparison of the intervention arm with the control arm

- Correlation of preoperative staging with postoperative pathological staging

- Rate of failure of the Oncotype DX® assay (RS result cannot be issued) on diagnostic core biopsy specimen

3.4 STUDY FLOW CHART

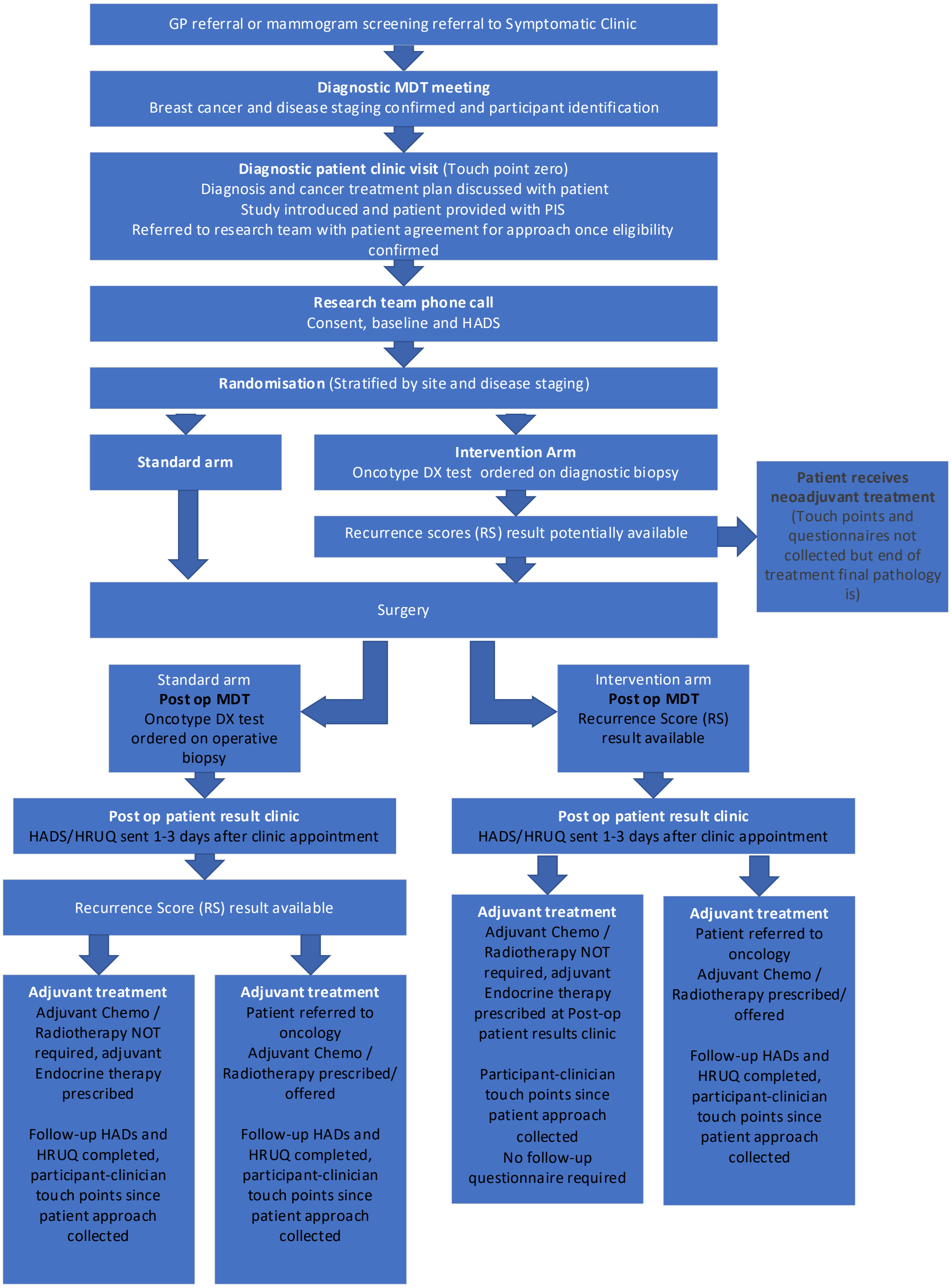

4.0 RECRUITMENT PROCESS

4.1 ELIGIBILITY

4.1.1. INCLUSION CRITERIA

- Male or female ≥ 18 years of age
- Oestrogen receptor positive HER2 negative invasive early stage breast cancer confirmed on diagnostic core biopsy, as per definition of hormone receptor status in NICE DG34 guidance
- Pre-operative staging of grade 2 cancer ≥ 20 mm on all imaging modalities or grade 3 cancer of any size and N0 on axillary staging or any size or grade which is N1 on preoperative axillary ultrasound scan +/- FNA or core biopsy*
- Surgery planned as first definitive treatment
- Fit for adjuvant chemotherapy
- Able to provide written or remote (eConsent or postal) informed consent
- Able to complete questionnaires and study assessments

* If local reimbursement available for N1 patients oncotype

4.1.2 EXCLUSION CRITERIA

- ER negative or HER2 positive breast cancer
- N2 disease on pre-operative staging
- Planned for neo-adjuvant systemic therapy (endocrine bridging therapy for a delay in planned surgery is permitted)
- Unfit for surgical treatment or systemic chemotherapy
- Are unable to provide informed consent
- Have co-existing malignant disease only if this would affect the study in the investigator's opinion
- Are unable to complete study questionnaires even with the assistance of the study nurse
- Are already participating in another clinical trial

4.2 IDENTIFICATION AND APPROACH

Patients will be referred to the breast unit following a GP referral or a suspicious mammogram screening result, and standard diagnostic screening tests completed as routine care. Patient cases will be discussed in the diagnostic MDT meeting, consisting of oncologists, surgeons, radiologists, pathologists and breast care nurses where the pre-test patient management plan will be determined. Breast cancer and disease staging will be confirmed and potential participants suitable for the study will be identified.

Potential participants will be seen in a pre-operative diagnostic clinic visit where their treating clinician will inform them of their cancer diagnosis. The treating clinician will discuss the study with the patient. If interested, they will be given a study Participant

Information Sheet (PIS) and a copy of the consent form to take away to consider. If all the required test results have been received and eligibility confirmed the potential participant, with their permission, will then be referred to the site research team.

If any results required to confirm eligibility are outstanding (e.g. HER2) at the diagnostic clinic appointment, the process as above can still be followed provided it is clear that their eligibility is dependent on any outstanding test results. The potential participant will only be approached by the site research team once eligibility is confirmed. Alternatively, the information (e.g. PIS) can be posted to the patient following the appointment along with the accompanying invitation letter ahead of eligibility being confirmed.

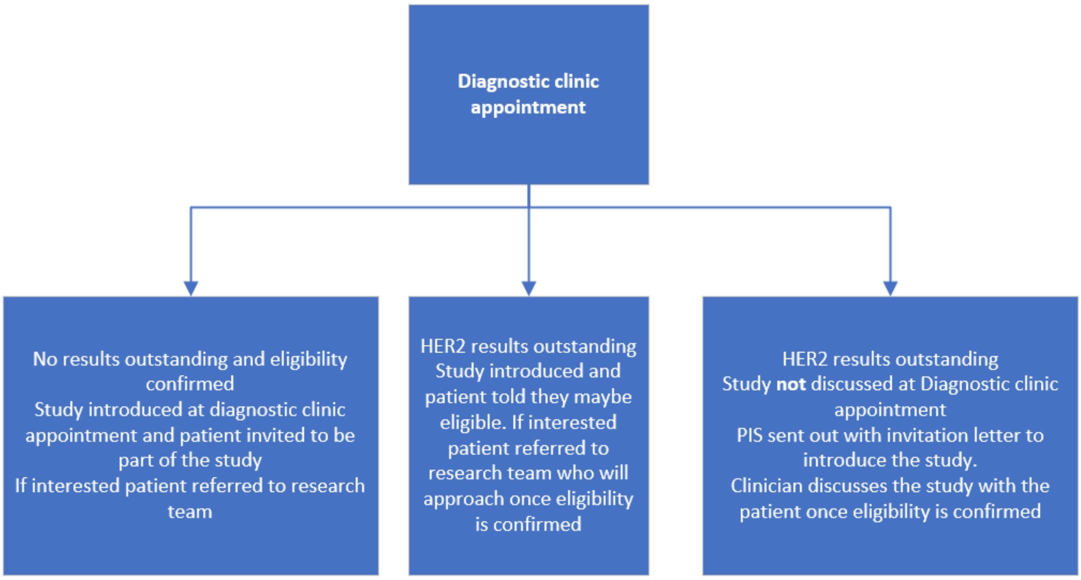

Figure 1. Pathways to patient approach

4.3 INFORMED CONSENT

Provided permission from the patient has been received, once eligibility has been confirmed the site research team will telephone or see the potential participant at the next appointment. This will occur no earlier than 24 hours after the PIS was provided to allow time for it to be read. The site research team will answer any questions the patient might have and if they are still interested in participating, will proceed to take informed consent. Consenting will be primarily be by remote consent, however, if the potential participant is scheduled to attend a planned follow-up clinic, the informed consent process may be conducted face to face. Informed consent will be taken by a study clinician or research nurse if authorised to do so on the delegation log and documented in the participants medical records.

For remote consent, either e-consent using DocuSign® or postal consent, if they are unwilling or are unable to access the internet, will be taken following real time discussion. In the event of postal consent being used, provided the participant has

confirmed that they have signed during the call, this will be taken as the date of consent. Due to the potentially tight study timelines, baseline assessment will be allowed to be completed before the consent form is received back and countersigned by the delegated site researcher. The participant returning the form at the next clinical appointment will be acceptable. For informed consent taken during face to face clinics either paper or e-consent methods will be used.

A copy of the consent form will be uploaded into the study REDCap Cloud data capture system (RCC) for monitoring purposes. The identity of the participant (if unknown to the site research team at the time of consent) will be checked at the next face to face clinic appointment to verify that the person that consented is who they say they are.

The study team will inform the participant's GP and clinical care team of their participation.

Study documents will not be translated into other languages. However, the use of hospital translation services will be permitted to support inclusion from all demographic groups.

Reasons for not taking part in the study will be closely monitored by Hull Health Trials Unit (HHTU). Participants do not have to provide a reason for their decision.

The PI has overall responsibility for informed consent of participants at site and must ensure that anyone delegated responsibility to conduct informed consent is duly authorised, trained and competent to undertake consent according to the protocol and Good Clinical Practice (GCP).

Once consented, the participant will then complete the baseline assessment which includes the Hospital Anxiety and Depression Scale (HADS) before being randomised.

4.3.1 PARTICIPANTS UNABLE TO GIVE CONSENT

For participants unable to read/sign the consent form, and for those with problems of comprehension (in accordance with the Mental Capacity Act 2005), study participation and informed consent will not be sought.

4.3.2 WITHDRAWAL OF PARTICIPANTS

Participants remain free to withdraw at any time from the study without giving reasons and without prejudicing their further treatment. During discussions around the participants withdrawal, they should be provided the option of withdrawing fully or just from the study questionnaires (e.g. no further contact with study team but touch points still recorded).
If the participant is willing to provide a reason for the withdrawal, this should be recorded when completing the withdrawal on the study database, although the participants' right not to give a reason must be respected throughout. Data collected up

to the point of participant withdrawal will be included in data analysis but no further contact will be made with the participant.

The informed consent form includes a statement on the use of data after a participant withdraws from the study.

4.4 ENROLMENT AND RANDOMISATION

A purpose built, secure web-based data capture system with integrated randomisation will provided and maintained by HHTU. A HHTU statistician, independent from the study team, will prepare a randomisation schedule. Patients will be randomised to Intervention and Standard Arm in 2:1 ratio using random permuted blocks, stratified by site and disease staging to balance potential confounding factors. The standard clinical care pathway at participating sites will introduce some variation in terms of number of patient clinical touch points. Disease staging may alter the complexity and choice of treatment patients receive, and therefore the number of clinical touch points. It is therefore important to stratify randomisation by both site and disease staging. As this is not a blinded study, research teams and participants will know the study arm to which they have been allocated.

When a participant is enrolled on RCC, a Subject ID Number will be automatically generated that will be entered on all study documents from that point onwards. Prior to randomisation, the study team will be asked to confirm informed consent has been taken. Participants will then be randomised to Standard or Intervention arms with the site receiving immediate notification of allocation. HHTU will receive an automatic email notification to flag that a randomisation event has occurred.

Authorised trial staff with delegated responsibility for randomising patients will access the secure online randomisation system administered by HHTU. User accounts will be set up for delegated site staff by HHTU once all the necessary trial documentation and approvals for participation in the study have been received. Randomisation will not be activated at individual sites until the site green light has been confirmed.

5.0 DATA COLLECTION

5.1 STUDY VISIT SCHEDULE

The study visit schedule will follow routine clinical care pathways – the only additional non-routine visit will be a telephone or video call to obtain informed consent and conduct baseline questionnaires. There will be no change to participant standard pre-operative investigations or clinic attendances, and these will be conducted either remotely or Face to Face, depending on current practice at site and following current guidance for COVID-secure appointments

Visits will be scheduled by the treating clinicians according to site clinical care pathway protocols and as such there is no visit window for any of the visits. Informed consent must be taken before surgery so that participants can be randomised into the standard or intervention arm.

Data on the time taken and the number of clinical touchpoints/visits from the initial patient approach to the offer of adjuvant cancer treatment by both groups will be collected as a study outcome. At baseline, participants will complete the Hospital and Anxiety Scale (HADS) as part of the baseline assessment. The HADS and a health resource utilisation questionnaire will then be completed following the post-operative clinic and following the offer of adjuvant treatment. In the event of the two follow-up events being the same date only the following the post-operative clinic questionnaires will be completed.

5.2 DATA COLLECTION METHODS

Hospital data collected by the hospital will be recorded on electronic Case Report Forms (eCRFs) designed specifically for this study in the data capture system. Source data will be documented in medical records. Patient reported outcomes (HADS and HRUQ) will be captured using versions of the questionnaires either sent directly from the database, completed over the phone with the research nurse or by postal questionnaire sent by the site. Given the short available time window between the post-operative clinic and the RS being received, questionnaires should be sent within 3 days of the post-operative clinic appointment date of the initial surgery where possible. If no response has been received within one week ± 3 days of the questionnaire being sent, the patient will be followed up by phone. Participants will be provided with a patient diary to record any health care interactions to aid in the HRUQ completion. Only data relevant to this study will be collected. Data collected in the CRFs will be pseudo anonymised and will not identify the participant. The Subject ID Number will link the CRFs to the source data during monitoring visits.

Table of data collection

**Baseline data**	○ Consent<br>○ Demographics (year of birth, age, sex, ethnicity)<br>○ Histology / radiology / pathology reports on diagnostic investigations<br>○ Disease staging – TNM grading<br>○ HADS<br>○ Randomisation
**Post consent**	○ Oncotype DX® test date ordered on diagnostic biopsy (Intervention Arm only)
**Surgery**	○ Results of surgery – histology, radiology, pathology reports
**Post-op results clinic**	○ Oncotype DX® test date ordered (Standard Arm only) RS results delivered (intervention)
**One week after Post-op Clinic (± 4 days)**	○ HADS and HRUQ
**~2 weeks after Post-op clinic**	○ RS results delivered (Standard arm only)
**Adjuvant treatment offer and prescription**	○ Treatment type (specifics not required as not collecting treatment outcome data)<br>○ HADS and HRUQ<br>○ Number of clinician-participant interactions and participant touch points* (Patient approach to the offer and prescription of the first adjuvant treatment)

** Appendix A for definitions*

6.0 SAMPLE COLLECTION

There will be no study specific research samples collected. The diagnostic and surgical core biopsies documented in the study protocol are taken in routine clinical practice and will be collected and processed following standard local site protocols.

No additional samples will be retained for study purposes but consent will be sought for permission for further possible research testing on the biopsy tissue that is retained as part of standard clinical practice.

7.0 MONITORING

A risk-based approach to monitoring will be adopted for PRE-DX. It is anticipated monitoring will be predominantly through a combination of central and remote monitoring. On-site monitoring is not expected unless an escalation is triggered as a result of the central or remote monitoring. A HHTU Study Monitoring Plan will be developed and agreed by the sponsor, CI and Trial Management Group (TMG).

A Data Monitoring Plan will be agreed by the sponsor, CI and statistician to provide detailed instructions and guidance relevant to database set up, data entry, validation, review, query generation and resolution, quality control processes involving data access and transfer of data to the sponsor at the end of the study and archiving. All the information obtained about participants in the course of the study is confidential and will be held in accordance with the General Data Protection Regulation (GDPR 2018).

Data will be monitored for quality and completeness by the HHTU. Missing data will be chased until it is received or confirmed as unavailable. HHTU will reserve the right to intermittently conduct CRF to database and source data verification to CRF exercises on a sample of participants. Source data verification may involve direct access to participant medical records at participating sites.

8.0 SAFETY REPORTING

As this study collects data on routine interventions, there are no study specific assessments that will affect study safety. Therefore, this study will not collect any adverse event or serious adverse event data. Should an Investigator have any concerns regarding participant safety as an outcome of their participation in the trial, they should contact the Trial Management Team (pre-dx@hyms.ac.uk) and Chief Investigator (henry.cain@nhs.net) to raise these concerns as soon as possible.

The TMG will be kept informed of diagnosis and treatment outcomes in regular study oversight meetings. Any trends that point towards patient harm will be identified and acted upon.

9.0 STUDY OVERSIGHT RESPONSIBILITIES

9.1 DAY-TO-DAY TRIAL MANAGEMENT

Day-to-day study management will be conducted by the HHTU study team who will work closely with the CI and sponsor. The HHTU study team will include a Trial Manager, Trial Coordinator, a Data Manager and a Trial Administrator with quality assurance and study oversight provided by members of the HHTU Senior Management Team. Study activities will be performed according to the relevant HHTU / Sponsor Standard Operating Procedures (SOPs).

9.2 TRIAL MANAGEMENT GROUP (TMG)

The TMG will comprise the CI, co-investigators, Sponsor representatives, HHTU personnel and lay representatives. The TMG will meet regularly until study recruitment begins and during the first three months of recruitment. Thereafter, the TMG will meet quarterly if progress is satisfactory and more frequently if not. Each meeting of the TMG will review achievement of milestones, rate of participant recruitment, data collection and safety issues. The need for an independent Trial Steering Committee was assessed by the Sponsor as part of the study risk assessment. As the study does not alter patient treatment, with no expected impact on patient safety, it was agreed that the TMG could conduct the oversight role for the study. If implementation challenges are identified, solutions will be sought and changes put in place. All TMG discussions will be minuted, with actions detailed, and reviewed at the following meeting.

If the risk profile of the study changes during the course of the study the need for an independent TSC would be reviewed.

10.0 COST IMPACT ANALYSIS

This component of work aims to evaluate health system cost impacts from any potential streamlining of the patient management pathway due to the availability of the RS® results from the core biopsy in the pre-operative setting.

The hypothesis is that the availability of RS results from the core biopsy in the pre-operative setting will reduce healthcare utilisation between diagnosis and the commencement of adjuvant treatment. To evaluate this, the study will collect data on: i) the number of health care provider-participant touch points throughout the follow-up, and ii) the conduct of repeat testing (including cases where there is failure of the Oncotype DX® assay on core biopsy specimen and re-testing proceeds using the surgical specimen. Furthermore, a Health Resource Utilisation questionnaire (HRUQ) will be developed and applied at various data collection time points throughout the study. This will aim to identify any further impacts on health care resource use, for example, relating to contacts with primary care providers.

The resource utilization will then be costed using appropriate sources, including NHS reference costs and national tariffs.

Difference in resource use between the two arms of the trial will be descriptively summarised. Difference in total costs will be examined by linear regression controlling for the same stratification factors and prognostic covariates as the statistical analyses.

11.0 STATISTICAL ANALYSIS PLAN

11.1 SAMPLE SIZE:

A single retrospective cohort study in the UK demonstrated that the earlier availability of the RS results, when ordered from the post op MDT, reduced the average number of post-operative clinical appointments by 1 visit [5]. We hypothesise that the number of participant-clinician touch points in the control group is 6 visits per patient and our intervention is likely to reduce it to 5 visits per patient. With 2:1 recruitment ratio between intervention and control group and 20% dropout rate, a sample size of 330 in total is required (220 in intervention arm and 110 in control arm). This sample size was calculated by the sample size and power calculation software, nQuery [6], for comparison of numbers of touch points between intervention and standard arm using a Negative Binomial model, at 5% significance level and 90% power– see Appendix B for the screenshot of sample size calculation.

11.2 STATISTICAL ANALYSIS:

The reporting guideline of CONSORT 2010 statement [7] will be followed for analysis and reporting of this study. All statistical tests and confidence intervals will be two-sided.

Ninety-five percent confidence intervals will be presented with the significance of p value at 5% significance level. Analyses will be conducted on an intention-to-treat basis (all participants who are randomized are included in the statistical analysis, including those who fail to receive an RS result from either the diagnostic core biopsy or excision specimen) and follow a pre-specified statistical analysis plan. Baseline patient characteristics will be tabulated through descriptive statistics. For the primary outcome, comparison of the numbers of participant-clinician touch points will be undertaken using a Negative Binomial model, adjusting for stratification factors and other prognostic baseline covariates. Rate of alteration in treatment pathway and rate of failure of obtaining an RS result on diagnostic core biopsy specimen in the intervention arm will be summarised and a per-protocol analysis will be conducted as secondary analysis, excluding those with alteration in treatment pathway or failure to obtain RS result. Comparison of time between diagnosis and adjuvant treatment will be assessed via Cox regression controlling for the same stratification factors and prognostic covariates. HADS score at baseline, the post-operative result clinic and commencement of adjuvant treatment will be examined by linear regression controlling for the same stratification factors and prognostic covariates. Correlation of preoperative staging with postoperative pathological staging will be calculated.

12.0 DATA SYSTEMS

Data will be hosted and managed by the Hull Health Trials Unit (HHTU) using their secure online data capture system REDCap Cloud (RCC), BOX Governance file storage system and esignature platform DocuSign. HHTU hold an NHS Digital Data Security and Protection Toolkit covering these information systems.

The HHTU Box is a cloud content management system which complies with (and is independently audited against): ISO27001/ISO27018 certified and holds SOC1,2 and 3 reports. Box use IBM servers, the primary data centre is London with a backup in Frankfurt. Access is limited to HHTU staff and authorised members of the research team.

RCC is a cloud-based EDC system provided by nPhase. Data is stored on dedicated RCC hardware in EU data centres (including real-time backup) managed by Amazon Web Services to industry standards outlined in ISO 27001, PCI DSS, SOC 1 - 3, FISMA, CIS, CSA, NIST and UK Cloud Security Principles. Data is encrypted at rest and in transit. RCC deliver compliance to HIPAA, CFR Part 11, and EMEA Annex 11.

DocuSign is a cloud-based eSignature provider. The HHTU instance is managed solely by HHTU staff and is within scope of the HHTU's NHS Data Security and Protection Toolkit. DocuSign holds ISO27001, SOC1 and SOC2, reports and has EU approval for their Binding Corporate Rules. Data is encrypted at rest and in transit using AES encryption and TLS1.2 with 256 bit keys and stored in EU data centres. Under GDPR DocuSign act as a data processor on behalf of the University of Hull who is the data controller for HHTU projects. DocuSign uses user specific login, logically separating access to documents meaning only staff working on a project have access to the data

All of these systems have been through rigorous Information Governance and Security assessments during the University of Hull's procurement process. Data is encrypted at rest and in transit.

13.0 DATA ACCESS

Access to the EDC system is managed by HHTU staff who will create users on behalf of the study team. Users will be required to complete training and sign a terms of use document. Users are granted role-based access which will be limited only to the sites they are working at. All activity within RCC is subject to full user audit including timestamp.

RCC restricts access to authorised study personnel and can assign individual access levels as appropriate using granular permissions. HHTU will ensure no direct patient identifiers are present in any exported datasets. RCC audit reports will be included as a standing agenda item within the TMG meetings to ensure regular review. The study will be assigned a dedicated Data Manager from the HHTU to oversee and monitor the conduct of the database. As part of the development process full specifications are generated for the study database listing each variable, eCRF and how they fit together into different time points (events) within the patient pathway. Development includes different environments for build, test and live, creating full validation records. RCC supports automated checks on data entry appropriate to the question data type. These can either be: ‘hard’ checks where the form cannot be saved with the value entered or soft validations where the form can be saved but a query is raised which sites can respond to through the system. A combination of these checks will be specified, developed and tested as part of the controlled database development process. RCC supports manual querying of data on the system. Queries are notified to site who will respond the through the system. This will be used by HHTU to raise appropriate queries on data that cannot be monitored through automated checks.

RCC supports labelling some fields as mandatory and others as optional. Where a field is mandatory the eCRF cannot be marked as complete and locked until there is data in it. These eCRFs will be chased by the HHTU as part of the ongoing data management process. The study dataset will be exported from RCC for analysis and stored in HHTU Box instance with restricted access. HHTU SOPs will be followed for the process of database lock and export including pseudo-anonymisation.

14.0 QUALITY ASSURANCE AND ETHICAL CONSIDERATIONS

The study will be conducted in accordance with the principles of Good Clinical Practice (GCP) as applicable under UK regulations, the NHS Research Governance Framework (RGF), and through adherence to Standard Operating Procedures (SOPs).

The HHTU study team will oversee study monitoring activities and ensure that the study is conducted in line with agreed SOPs. HHTU will conduct internal audits on study management at least annually.

14.1 RESEARCH ETHICS COMMITTEE (REC)

Before the start of the study, approval will be sought from a REC for the study protocol, informed consent forms and other relevant participant facing documents. Substantial Amendments that require review by REC will not be implemented until the REC grants a favourable opinion for the study (note that amendments also need to be reviewed and accepted by NHS R&D departments before they can be implemented in practice at sites). All correspondence with the REC will be retained in the Trial Master File/Investigator Site File. An annual progress report (APR) will be submitted to the REC within 30 days of the anniversary date on which the favourable opinion was given, and annually until the trial is declared ended. The Chief Investigator will be responsible for the annual and end of study reports as required.

14.2 REGULATORY COMPLIANCE

Before any site can enrol patients into the trial, the CI/PI or designee will apply for NHS HRA permission and complete a Capacity and Capability assessment with site Research & Development (R&D) departments. For any amendment that will potentially affect sites NHS permissions, the CI/PI or designee will confirm with that site R&D department that NHS permission is ongoing (note that both substantial amendments and amendments considered to be non-substantial for the purposes of REC may still need to be notified to NHS R&D).

14.3 PROTOCOL COMPLIANCE

Protocol deviations, non-compliances, or breaches are departures from the approved protocol. Prospective, planned deviations or waivers to the protocol are not allowed under the UK regulations on Clinical Trials and will not be used e.g. patients will not be enrolled if they do not meet the eligibility criteria or restrictions specified in the study protocol. Accidental protocol deviations can happen at any time. They will be adequately documented on the relevant forms and reported to the CI and Sponsor immediately. Deviations from the protocol which are found to frequently recur may trigger additional monitoring visits and refresher training sessions. They may be classified as a serious breach.

14.4 SERIOUS BREACHES

HHTU and CIs are required to promptly notify the sponsor of a potential serious breach (as defined in the latest version of the National Research Ethics Service (NRES). A 'serious breach' is defined as a breach of the protocol or of the conditions or principles of GCP (or equivalent standards for conduct of non-CTIMPs) which is likely to affect to a significant degree:

(a) the safety or physical or mental integrity of the trial subjects
(b) the scientific value of the research

In the event of a serious breach the sponsor will be notified immediately of any case where the above definition applies during the trial conduct phase. The sponsor will notify the licensing authority of any serious breach of:

(a) the conditions and principles of GCP in connection with that trial

(b) the protocol relating to that trial, as amended from time to time, within 7 days of becoming aware of that breach

In the event of doubt or for further information on whether a protocol deviation should be classed as a serious breach, the PI should contact the Trial Manager at the HHTU.

14.5 ETHICAL CONSIDERATIONS

The study will be performed in accordance with the recommendations guiding physicians in biomedical research involving human subjects adopted by the 18th World Medical Assembly, Helsinki, Finland, 1996, amended at the 52nd World Medical Association General Assembly, Edinburgh, Scotland.

Written Informed consent will be obtained from participants prior to study randomisation/registration.

The right to decline study participation without giving a reason must be respected. The participant must remain free to withdraw at any time without giving a reason and without prejudicing his/her further treatment.

The study will be submitted to and approved by a main REC and each site R&D team prior to entering participants into the study. The HHTU will provide the main REC with a copy of the final protocol, participant information sheets, consent forms and all other relevant study documentation.

15.0 CONFIDENTIALITY

All information collected during the course of the study will be kept strictly confidential. Information will be held securely either on paper and / or electronically at site and at HHTU. HHTU will comply with all aspects of GDPR 2018. Operationally this will include:

- Consent from participants to record personal details: name, date of birth, address, telephone number, NHS number, hospital number, GP name and address
- Appropriate storage, restricted access and disposal arrangements for participant personal and clinical details
- Consent from participants for access to their medical records by responsible individuals from the research staff or from regulatory authorities, where it is relevant to study participation

- Consent from participants for the data collected for the study to be used to evaluate safety and develop new research
- Data collection forms that are sent to the HHTU for remote monitoring purposes will be coded only with a Subject ID Number. No personal identifiers will be documented
- Where central monitoring of source documents by HHTU (or copies of source documents) is required (such as biopsy results, clinical records), the participants name must be redacted by site before sending
- Where anonymisation of documentation is required, sites are responsible for ensuring only the instructed identifiers are present before sending to HHTU

16.0 ARCHIVING

The archiving of study documents and data at site will be done according to Trust policies and SOPs and will be retained for a period of 5 years. HHTU will archive TMF and datasets in line with HHTU / sponsor SOPs.

17.0 STATEMENT OF INDEMNITY

A statement of indemnity will be filed in the Trial Master File for HHTU and sponsor sites. Sites that include a member of staff with an honorary contract, will provide a copy of the honorary contract to HHTU.

18.0 STUDY ORGANISATIONAL STRUCTURE

Chief Investigator (CI) – The CI will have overall responsibility for the trial design, set-up, conduct and co-ordination and management.

Trial Sponsor – The Sponsor will be responsible for trial initiation management and financing of the trial as defined by Directive 2001/20/EC. These responsibilities are delegated to the HHTU as detailed in the trial contract.

Hull Health Trials Unit – HHTU will have responsibility for study conduct as delegated by the Sponsor in accordance with GCP standards. HHTU will conduct study set-up and monitoring in line with HHTU SOPs, partner SOPs (if applicable) and the UK Policy Framework for Health and Social Care Research. Responsibilities include: study administration, protocol development, CRF design, study design, main REC/HRA regulatory submissions, data management, safety reporting, randomisation design and service, database development and provision, database administrative functions, source data verification, monitoring, statistical analyses, HHTU and site training, study reports and results dissemination

Trial Management Group (TMG) – A TMG Terms of Reference document will be developed and approved at the first meeting. The TMG will be responsible for oversight of study set-up, study management, study data collection, safety issues, data quality and analysis and publishing study results.

19.0 PUBLICATION POLICY

The contract between Exact Science and the other parties will outline the publication policy, which will be in line with the Publication policy at Exact Sciences and mutually agreeable to all parties. A HHTU general approach to publication policy is given here and will be updated in later amendments to reflect the wording used in the contract. It is not expected to be greatly different in principle to that given below.

Prior to study recruitment, the study will be registered with an authorised registry, according to the International Committee of Medical Journal Editors (ICMJE) Guidelines.

The success of the study depends upon the collaboration of all participants. For this reason, credit for the main results will be given to all those who have collaborated on the study, through authorship and contributions. Authorship guidelines will be provided for manuscripts submitted to medical journals. These state that authorship credit should be based only on substantial contribution to:

- conception and design, or acquisition of data, or analysis and interpretation of data
- drafting the article or revising it critically for important intellectual content
- and final approval of the version to be published
- and that all these conditions must be met (www.icmje.org)

The CI and relevant senior HHTU staff will be named as authors in all publications. All collaborators will be listed as contributors for the main study publication, alongside roles in study planning, conducting and reporting. To maintain study scientific integrity, data will not be released before the first publication of primary endpoint analysis, either for publication or oral presentation, without TMG permission. Individual collaborators must not publish data concerning their participants before the first publication of primary endpoint analysis.

20.0 REFERENCES

1. *NICE Guidance DG34. . Available from: https://www.nice.org.uk/guidance/dg34/chapter/1-Recommendations.*
2. *Sparano, J.A., et al., Adjuvant Chemotherapy Guided by a 21-Gene Expression Assay in Breast Cancer. New England Journal of Medicine, 2018. **379**(2): p. 111-121.*
3. *Anderson, J., et al., Molecular Characterization of Breast Cancer Core Biopsy Specimens by Gene Expression Analysis Using Standardized Quantitative RT-PCR, in Poster Session Abstracts. 2009. p. 6021-6021.*
4. *Jakubowski, D.M., et al., Molecular characterization of breast cancer needle core biopsy specimens by the 21-gene Breast Recurrence Score test. Journal of Surgical Oncology, 2020. **122**(4): p. 611-618.*
5. *Yarwood, A., et al., Evolution of the post-surgical breast cancer pathway for adjuvant treatments following the introduction of genomic profiling for selected women with hormone reseptor positivebreast cancer. . 2020, Presented at UK Interdisciplinary Breast Cancer Symposium January 2020.*

6. *nQuery, Sample Size and Power Calculation. Version 8.4.1.0. "Statsols" (Statistical Solutions Ltd), Cork, Ireland. . 2017.*
7. *Schulz KF, Altman DG, Moher D, for the CONSORT Group. CONSORT 2010 Statement: updated guidelines for reporting parallel group randomised trials. Ann Int Med 2010;152. Epub 24 March.*
8. *Ralph et al, UK Interdisciplinary Breast Cancer Symposium 2020. Breast Cancer Res Treat 180, 527–596 (2020). https://doi.org/10.1007/s10549-019-05514-3*

APPENDIX A - DEFINITIONS OF PARTICIPANT-CLINICIAN INTERACTIONS AND PARTICIPANT TOUCH POINTS

The list provides examples of clinical interactions (participant-clinician) and participant touch points.

Participant approach is touchpoint zero, all other touchpoints from that point onwards until the first adjuvant treatment is offered and prescribed are counted.

**Hospital reported Participant-Clinician Interactions – primary outcome**	**Participant and hospital reported healthcare touchpoints – health economic outcomes**
Oncology Clinician outpatient visits or equivalent	As Hospital reported plus:
Oncology nursing outpatient visits or equivalent	
Surgical clinician outpatient visits or equivalent	GP visits
Surgical nursing outpatient or equivalent	GP Practice Nurse visits (home)
Radiology clinician outpatient visits or equivalent	GP Practice Nurse visits (surgery)
Radiology nursing outpatient visits or equivalent	A&E visits related to breast care
Other breast care related secondary care visits or equivalent	

APPENDIX B – SCREENSHOT OF SAMPLE SIZE CALCULATION

With dispersion parameter set as 0, 88 and 176 participants are needed for the final analysis. To accommodate 20% dropout rate, this leads to 110 and 220 participants to recruit.

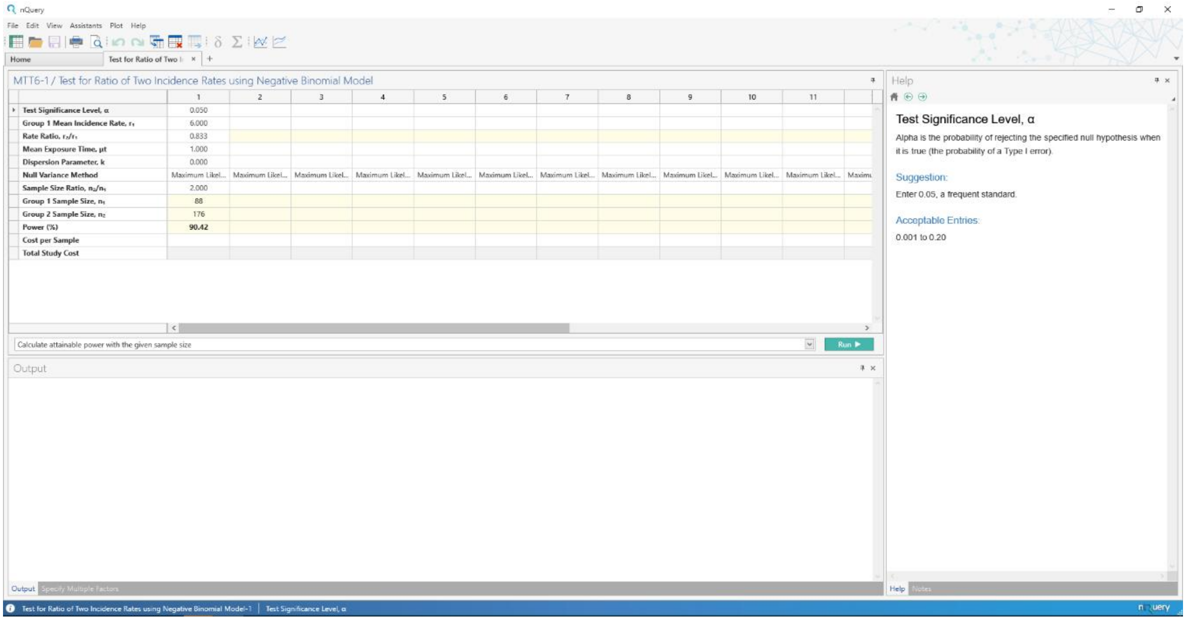
nQuery
File Edit View Assistants Plot Help
Home
Test for Ratio of Two I
MTT6-1 / Test for Ratio of Two Incidence Rates using Negative Binomial Model
Test Significance Level, α 0.050
Group 1 Mean Incidence Rate, r1 6.000
Rate Ratio, r2/r1 0.833
Mean Exposure Time, μt 1.000
Dispersion Parameter, k 0.000
Null Variance Method Maximum Likel...
Sample Size Ratio, n2/n1 2.000
Group 1 Sample Size, n1 88
Group 2 Sample Size, n2 176
Power (%) 90.42
Cost per Sample
Total Study Cost
Calculate attainable power with the given sample size
Run
Output
Help
Test Significance Level, α
Alpha is the probability of rejecting the specified null hypothesis when it is true (the probability of a Type I error).
Suggestion:
Enter 0.05, a frequent standard.
Acceptable Entries:
0.001 to 0.20
Test for Ratio of Two Incidence Rates using Negative Binomial Model-1
Test Significance Level, α
