# Supplementary material for: A randomised controlled trial of Pre-Operative Oncotype DX testing in early-stage breast cancer (PRE-DX study) – Study protocol
Source: PLoS One. 2024 Mar 15;19(3):e0300339. doi: 10.1371/journal.pone.0300339 (PMC10942020; doi:10.1371/journal.pone.0300339)
Supplement: Attachment — Submitted filename: PONE-D-23-30937 Response to reviewers .docx [file pone.0300339.s003.docx]

PONE-D-23-30937

A Randomised Control trial of Pre-Operative Oncotype DX® Testing in early-stage breast cancer  – Study Protocol

PLOS ONE

Dear Dr Daniele Ugo Tari,

Thank you for providing us with the reviewers’ comments on our paper entitled: A Randomised Controlled trial of Pre-Operative Oncotype DX® Testing in early-stage breast cancer  – Study Protocol.

We have addressed all of the comments as detailed below (page numbers given refer to page numbers on the revised manuscript with tracked changes). To incorporate the suggested changes we have made some other minor changes to keep the manuscript within the required word limit.

We would like to thank all three reviewers for their helpful comments.

First, we will address the journal requirements before responding to the reviewer comments.

1. Please ensure that your manuscript meets PLOS ONE's style requirements, including those for file naming. The PLOS ONE style templates can be found at

<https://journals.plos.org/plosone/s/file?id=wjVg/PLOSOne_formatting_sample_main_body.pdf> and 
<https://journals.plos.org/plosone/s/file?id=ba62/PLOSOne_formatting_sample_title_authors_affiliations.pdf>

**Response**

We have corrected the author addresses as per the title page formatting guidance, removing the post code from affiliation address 9 (Pankaj Roy) – Lines 21-22, the street and building from affiliation address 10 (Sarah Reynia) – Line 23, and street from affiliation address 12 (Henry Cain) -lines 25-26.

The citing and presentation of titles for all figures have been corrected to comply with the formatting guidance for the main body of text.

In addition, the file naming has been corrected (Lines 119-120, 246, 249).

2. Please note that funding information should not appear in any section or other areas of your manuscript. We will only publish funding information present in the Funding Statement section of the online submission form. Please remove any funding-related text from the manuscript.

**Response**

We have removed the funding statement from the title page as per the title page formatting guidance.

3. Thank you for stating the following in the Competing Interests section:

"Matthew Northgraves has nothing to disclose.
Judith Cohen reports a grant from Exact Sciences, during the conduct of the study.
James Harvey reports a consultancy fee from Genomic Health.
Chao Huang has nothing to disclose.
 
Carlo Palmieri reports grants from Daiichi Sankyo, Gilead, Pfizer, Seagen, consulting fees from AstraZeneca, Daiichi Sankyo, Eli Lilly, Eisai, Exact Sciences, Gilead, Medac, MSD, Novartis, Pfizer, Roche,  Seagen, honoraria for presentations/educational events from Pfizer, AZ, Seagen, receipt of equipment from  Pfizer, Seagen, travel and meeting support from Gilead, Roche, Novartis and a role with the NCRI.

Sarah Pinder reports honoraria for presentations/educational events, travel support and participation on advisory boards and workshops for Exact Sciences.

Pankaj .G. Roy reports personal fees from Exact Sciences, outside the submitted work.
Sarah Reynia reports employment from, and stock in Exact Sciences.
Marta Soares has nothing to disclose.
 
Henry Cain reports a grant for the conduct of this study, honoraria for presentations/educational events, and consulting fees from Exact Sciences."

We note that you received funding from a commercial sources: Daiichi Sankyo, Gilead, Pfizer, Seagen, AstraZeneca, Eli Lilly, Eisai, Exact Sciences, Medac, MSD, Novartis, Roche & Genomic Health.

Please provide an amended Competing Interests Statement that explicitly states this commercial funder, along with any other relevant declarations relating to employment, consultancy, patents, products in development, marketed products, etc.

Within this Competing Interests Statement, please confirm that this does not alter your adherence to all PLOS ONE policies on sharing data and materials by including the following statement: ""This does not alter our adherence to PLOS ONE policies on sharing data and materials.” (as detailed online in our guide for authors <http://journals.plos.org/plosone/s/competing-interests>).

If there are restrictions on sharing of data and/or materials, please state these. Please note that we cannot proceed with consideration of your article until this information has been declared.

Please include your amended Competing Interests Statement within your cover letter. We will change the online submission form on your behalf.

**Response**

The following amended Competing Interest Statement has been added to the cover letter.

Matthew Northgraves, Chao Huang and Marta Soares have nothing to declare.

Judith Cohen reports a grant from commercial funder Exact Sciences for the conduct of the study.

Henry Cain reports a grant for the conduct of this study as well as, honoraria for presentations/educational events, and consulting fees from Exact Sciences."

James Harvey reports a consultancy fee from Genomic Health.

Carlo Palmieri reports consulting fees from Exact Sciences along with AstraZeneca, Daiichi Sankyo, Eli Lilly, Eisai, Exact Sciences, Gilead, Medac, MSD, Novartis, Pfizer, Roche, Seagen; grants from Daiichi Sankyo, Gilead, Pfizer, Seagen; honoraria for presentations/educational events from Pfizer, AZ, Seagen; receipt of equipment from  Pfizer, Seagen; travel and meeting support from Gilead, Roche, Novartis and a role with the NCRI.

Sarah Pinder reports honoraria for presentations/educational events, travel support and participation on advisory boards and workshops for Exact Sciences.

Pankaj .G. Roy reports personal fees from Exact Sciences, outside the submitted work.

Sarah Reynia reports employment from, and stock in Exact Sciences.

This does not alter our adherence to PLOS ONE policies on sharing data and materials.

4. Please amend either the title on the online submission form (via Edit Submission) or the title in the manuscript so that they are identical.

**Response**

The manuscript title has been amended to ‘*A Randomised Controlled trial of Pre-Operative Oncotype DX® Testing in early-stage breast cancer – Study Protocol’* (Line 1) and the online submission title has been edited accordingly so it now matches the manuscript as requested.

5. Please include captions for your Supporting Information files at the end of your manuscript, and update any in-text citations to match accordingly. Please see our Supporting Information guidelines for more information: <http://journals.plos.org/plosone/s/supporting-information>.

**Response**

Supporting information captions have been added to the end of the manuscript (Lines 426-428) and in-text citations included (lines 119-120 and 373) as requested.

6. We note that the original protocol that you have uploaded as a Supporting Information file contains an institutional logo. As this logo is likely copyrighted, we ask that you please remove it from this file and upload an updated version upon resubmission. 

Logos in the Original REC approved protocol have been removed as requested.

***Reviewer One comments:***

Reviewer #1: In this study protocol, a two-arm randomized (2:1) controlled multi-center trial is being proposed which aims to compare the impact on the patient treatment pathway of performing Oncotype DX test on the diagnostic core biopsy pre-operatively (intervention) to the excised invasive carcinoma (control). The primary endpoint is the number of clinical interactions between treating team and patient from initial approach until offer and prescription of the first adjuvant treatment.

Minor revisions:
1- Line 353: Clarify if Cox regression implies Cox proportional hazards regression.

**Response**

We can confirm that Cox proportional hazards regression will be used and the manuscript has been updated accordingly (line 320).

2- Line 357: State the statistical methods that will be used for correlating preoperative staging with postoperative pathological staging.

**Response**

We will use Spearman’s rank correlation for correlating preoperative staging with postoperative pathological staging. The manuscript has been updated accordingly (lines 325-326).

3- Identify the software that will be used for the statistical analysis.

**Response**

All statistical analyses will be performed using SPSS version 28 and R language version 4.2. The manuscript has been updated accordingly (lines 306-307).

***Reviewer two comments:***

Comments/Revisions
1. The impact of preoperative Oncotype testing in the management of early-stage breast cancer is worthy investigation and there is merit in conducting the proposed trial

**Response**

We would like to thank the reviewer for agreeing the topic is worthy of investigation and that there is merit in performing the proposed trial.

2. The main benefit of preoperative testing would be the reduction in time from diagnosis to adjuvant therapy. Therefore, I feel that time to adjuvant therapy should be the primary endpoint. Number of clinical touchpoints can be one of the secondary endpoints.

**Response**

We agree that reducing the time from diagnosis to adjuvant treatment is one of the main benefits of pre-operative oncotype testing. This was given due consideration during the development stage of the study; however, it was felt the reduction of clinical touchpoint was a more suitable primary outcome measure for the following reasons:

a) The COVID-19 pandemic caused considerable backlogs in NHS appointments and waiting times grew. As a result, some NHS trusts implemented a standard two to three months wait time for post-surgery oncology appointments. Had this been a single site study we would had been more confident of using time to surgery but due to the variability across multiple units in terms of waiting list, it was felt this may have diluted any potential benefits that may have been seen.
b) As discussed in the introduction, there is a large financial benefit to reducing the number of costly outpatients’ appointments. Current NHS National Tariff costs (2022/23 Annex A: The national tariff workbook) have consultant led follow-up Breast surgery outpatient appointment costing £79-£97 and clinical oncology outpatient at £132 - £147 per appointment. There would be a significant financial benefit to the NHS from removing unnecessary appointments whilst potentially reducing waiting times.
c) Reducing the number of hospital visits from a patient’s point of view is very important and highlights its value as a primary endpoint.

It would not be possible to change the primary endpoint at this point in the study, as recruitment and data collection are in progress. The trial was designed with the primary outcome as defined and the sample size calculated based on this. It is an important principle in the good conduct of clinical trials that the primary outcome is predefined before the study commences.

3. The agreement between preoperative and postoperative Oncotype should be reported.

**Response**

We wholeheartedly agree that the agreement between the preoperative and postoperative Oncotype is an important measure, however at the current time, we have not got the funding to enable analysis using paired sample testing. The Oncotype test was funded according to available NHS funding for clinical care and performed once at the time point dependent on randomised allocation. It would be possible to order the additional test on the pre or post-operative samples that are taken routinely, but the additional testing would need to be funded from the research funder. This element of the study has been presented to the funder as an additional sub-study and is still under consideration. A consent statement has also been included in the participant consent forms allowing the paired sample to be conducted if the funding is secured.

4. Professional editing is recommended prior to publication

**Response**

We unfortunately do not have the budget for professional editing.

***Reviewer three comments:***

Reviewer #3: I am very grateful to you for giving me the opportunity to review this interesting manuscript where the authors describe a new approach to the treatment of early breast cancer. The authors propose to perform the OncotypeDx on the biopsy sample of patients with surgical indication, instead of surgical specimen, in order to optimize the initiation of adjuvant treatment. However, I have a few concerns about the protocol:

1. The decision for adjuvant treatment is typically based on an evaluation of both clinical and genomic risk factors, as detailed by Sparano et al (N Engl J Med 2019; 380:2395-2405). Therefore, it is an interesting strategy to conduct the genomic analysis on the biopsy sample, ensuring that the initiation of adjuvant treatment is not delayed. Nevertheless, there are certain scenarios in which it is unnecessary to utilize the genomic platform to make decisions about adjuvant treatment. This includes cases involving premenopausal patients with positive axillary fine-needle aspiration (FNA) or tumors larger than 3 cm with grade 3. Performing genomic platforms in this context could potentially lead to unnecessary costs for the health system. I kindly suggest to the authors to consider and describe strategies in the protocol that could help minimize such scenarios during the study, with the aim of optimizing resource utilization.

**Response**

We agree with the reviewer’s comment that there are certain scenarios when clinically it is unnecessary to utilise the genomic platform as described. Recruiting sites were informed during the site set-up that only patients for whom the oncotype was clinically required to guide their treatment were eligible for the study. This message has been reinforced during the recruitment period at monitoring calls with sites. The test is ordered as part of the patients’ standard of care, and sites were informed that no tests should be ordered purely for the purpose of the research study. We acknowledge that there is the potential that tests ordered in the pre-operative setting may no longer be required in the post-operative setting (e.g. cancer discovered to be metastatic during surgery) and the frequency at which this occurs will be reported.

As this detail was not explicitly stated in the REC approved protocol we do not think that it would be appropriate to include the details in this manuscript. However, this will be reported and discussed in the results paper.

2. The authors describe the analysis of the number of contact points between the clinician and the participant as the primary outcome and calculate the sample size based on the reduction from 6 to 5 interactions. I kindly suggest that the authors provide an explanation in the manuscript regarding why this reduction might be considered relevant in the treatment of these patients. Additionally, the authors could discuss how this reduction might impact costs to the healthcare system. Providing such context would enhance the understanding of the significance of the chosen primary outcome and its implications for both patient care and resource utilization.

**Response**

Again, we thank the reviewer for these very valid suggestions. The reduction from 6 to 5 clinician and participant interactions came from pre-study modelling, based on typical pathways at the four breast units which have members on the trial management group. We agree discussion regarding why the reduction may be relevant to patients and more widely what the impact on resource utilization could be is important and will be included in the results paper. However, as stated above, as this detail was not explicitly stated in the REC approved protocol, we do not think that it would be appropriate to include the details in this manuscript.
